# Effects of mHealth Practice Patterns on Improving Metabolic Syndrome Using the Information–Motivation–Behavioral Skills Model †

**DOI:** 10.3390/nu16132099

**Published:** 2024-07-01

**Authors:** Na-Young Park, Sarang Jang

**Affiliations:** 1 Korea Institute for Health and Social Affairs, Sejong 30147, Republic of Korea; dudsky04@kihasa.re.kr; 2 Department of Public Health, Sahmyook University, Seoul 01795, Republic of Korea

**Keywords:** mHealth, IMB model, metabolic syndrome, chronic disease, health promotion, GBTM, GEE

## Abstract

Chronic diseases contribute to 68% of global mortality, highlighting the importance of early detection and management of conditions such as metabolic syndrome. Effective lifestyle interventions, particularly through mobile health (mHealth), have shown potential in promoting health and reducing cardiometabolic risk. This study utilized mHealth data from public health centers in South Korea, targeting adults with risk factors for metabolic syndrome. The Intervention-Motivation-Behavioral skills (IMB) theoretical model was applied to categorize participants’ practice patterns over time using the Group-Based Trend Model (GBTM). And the Generalized Estimating Equations (GEE) methodology was applied to confirm the effective practice patterns for improving metabolic syndrome. Data were collected over 24 weeks. The dataset encompasses life-log data capable of capturing changes in intervention, self-report surveys, and clinical measurements, all linked to personal identification keys and thereby integrated. Participants demonstrated improved health behaviors, with the healthy eating score increasing from 5.0 to 6.4 and physical activity rates rising from 41.5% to 59%. Health risk factors decreased significantly, with the mean number of risk factors dropping from 2.4 to 1.4. The percentage of subjects with three or more metabolic syndrome components decreased from 42.3% in the initial period to 19.2% in the final period. Practice patterns by IMB components were classified into three categories: continuous type, late decline type, and early decline type. Improvements in health behavior and metabolic syndrome were observed in the continuous type of each IMB component. The mHealth interventions were confirmed to be positively associated with improved health behavior and management of metabolic syndrome in the continuous practice patterns of IMB.

## 1. Introduction

### 1.1. Background

The importance of preventing chronic diseases is emphasized as 68% of global mortality is due to chronic diseases [1,2]. Chronic diseases related to metabolic syndrome include cardiovascular disease, diabetes, hypertension, obesity, and dyslipidemia [3,4,5,6,7,8]. Management of metabolic syndrome plays a central role in the prevention of chronic diseases. Therefore, early detection and management of metabolic syndrome are crucial in preventing the development of chronic diseases [7,9,10].

Metabolic syndrome is a condition in which three or more health risk factors, such as high blood pressure, high blood glucose, abdominal obesity, hypertriglyceridemia, and low high-density lipoprotein (HDL) cholesterol level, are clustered together. In clinical practice, the modified National Cholesterol Education Program Adult Treatment Panel III (ATP III) [4] and the International Diabetes Federation (IDF) [11] definitions have become the most widely utilized. And in relation to abdominal obesity, application standards differing by ethnicity and country were agreed upon. In South Korea, the NCEP-ATP III standard is used to determine the health risk factors for metabolic syndrome. High blood pressure is defined as systolic blood pressure of 130 mmHg or more, or diastolic blood pressure of 85 mmHg or more. High blood sugar is defined as fasting blood glucose of 100 mg/dL or more. Hypertriglyceridemia is defined as triglycerides of 150 mg/dL or more. Reduced HDL cholesterol is defined as less than 40 mg/dL for males and less than 50 mg/dL for females. Abdominal obesity refers to the Asian standards presented by the IDF, applying a clinical standard of waist circumference of 90 cm or more for males and 85 cm or more for females as the criteria for abdominal obesity in adults.

The prevalence of metabolic syndrome presents considerable measurement challenges, resulting in a scarcity of comprehensive global data [9]. According to a recent meta-analysis of global data, the prevalence of metabolic syndrome varied from 12.5% to 31.4% by region, but predominantly tended to increase in line with the country’s income level [12]. As reported by the IDF Diabetes Atlas [13], the global prevalence of diabetes was 8.8% (415 million individuals) in 2015, with projections indicating an increase to 10.4% (642 million individuals) by 2040. Given that metabolic syndrome is estimated to be approximately three times more common than diabetes, it is plausible to infer that metabolic syndrome affects roughly one-quarter of the global population, equating to over one billion individuals currently afflicted by metabolic syndrome worldwide [9]. In South Korea, 21.3% of individuals undergoing a health checkup in 2021, accounting for approximately 17 million people, were identified as having metabolic syndrome. Consequently, early detection and management of metabolic syndrome are crucial for preventing the onset of chronic diseases [14].

However, metabolic syndrome can worsen into a disease state due to the combined effects of multiple risk factors influenced by poor lifestyle habits. Metabolic syndrome can be managed through improvements in lifestyle [5,11,15,16]. Therefore, upon identifying abnormal findings for one or more health risk factors, individuals should actively evaluate and improve their lifestyle habits, including smoking cessation, moderating alcohol consumption, adopting healthier dietary patterns, weight management, and regular physical activity [1,9,17,18].

Mobile health (mHealth) was introduced as a behavioral intervention strategy to enhance healthcare delivery, addressing the constraints of in-person services and resources. In particular, the exponential growth of health management applications (apps) from a preventive standpoint and the evolution of interconnected devices have heightened the demand for health promotion programs aimed at managing lifestyle habits, such as diet, physical activity, and sleep, among healthy individuals, thereby accelerating innovation in health management services. The European Commission indicated that mHealth functions as a valuable tool for fostering behavioral change. According to the report, mHealth assists patients in transitioning from passive to active engagement, enabling patients to manage their health through apps and adhere to dietary and medication routines [19]. Indeed, mHealth has significant potential for promoting a healthy lifestyle [8,20].

Health behavior interventions aimed at improving lifestyle habits should utilize multiple intervention methods, simultaneously [21]. By facilitating the implementation of multiple behavioral interventions beyond conventional face-to-face services, mHealth offers opportunities for education, information dissemination, establishing personalized goals, monitoring, individualized counseling, motivational app prompts, incentives, gamification, and peer communication. This enables comprehensive integration of various behavior change techniques (BCTs). Technological advances not only reinforce the promotion and maintenance of patient behavior through mHealth but also enhance the interaction between service providers and recipients, resulting in improved service quality through restorative feedback and a positive influence on health outcomes [22,23]. mHealth behavioral interventions exhibit higher service completion rates than control groups, with an average completion rate of 83.3% in one study [24] and 79.6% in a review [25]. Furthermore, multiple studies have indicated that health management utilizing mHealth not only impacts healthy eating and physical activity but also leads to other alterations in health behaviors [8,16,20,21,26,27,28,29].

However, studies investigating the impact of mHealth based on theoretical frameworks of practice patterns are limited. Therefore, this study applied a theoretical health promotion model to evaluate the collective health promotion effect of metabolic syndrome healthcare services utilizing mHealth by categorizing the quantified practice patterns of users for each BCT.

### 1.2. Theoretical Framework 

**Figure 1:**
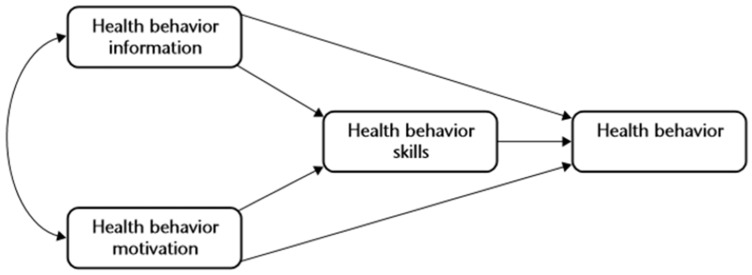
The Information–Motivation–Behavioral Skills model. Source: Fisher et al., 2003 [31].

Numerous theories have been proposed to elucidate health behavior changes, among which the Information–Motivation–Behavioral Skills (IMB) model is notable. This model conceptualizes the integration of information, motivation, and behavioral skills necessary to modify and sustain health-risk behaviors (Figure 1). 

According to the IMB model, individuals experience promoted behavior change and its maintenance, as well as positive health outcomes when they attain adequate health information, exhibit motivation for behavior change, and cultivate the requisite behavioral skills [30]. Information encompasses knowledge and essential technical details concerning the etiology, symptoms, treatment protocols, and actionable measures related to the symptom or disease. Motivation comprises an individual’s inclination to engage in health behaviors, the social support they receive, and subjective norms regarding health behaviors from their social environment, all of which exert significant influence on health behavior performance. Behavioral skills pertain to an individual’s tangible abilities and self-efficacy required for executing health-related actions. Thus, the IMB model enables the design and assessment of diverse intervention strategies and proves effective in health promotion and chronic disease management [31].

### 1.3. Objectives 

This study classifies various mHealth interventions according to the IMB theoretical model and categorizes participants’ practice patterns over time using the Group-Based Trend Model (GBTM). Additionally, this study aims to identify meaningful types of health-improvement effects from these categorized practice patterns. Furthermore, this study proposes an efficacious behavioral intervention method utilizing mHealth behavior change techniques (BCTs) for the management of metabolic syndrome.

## 2. Materials and Methods

### 2.1. Study Participants

This study utilized mHealth data from the public health centers of the Korea Health Promotion Institute to target adults with risk factors for metabolic syndrome. Launched as a pilot in 2016 and expanded annually, the program employed mobile phone apps and devices administered by a team of healthcare professionals to deliver tailored health services. This study analyzed mHealth data from 35 public health centers collected over 24 weeks from 15 May to 19 November 2017.

### 2.2. Contents of mHealth Intervention

**Figure 2:**
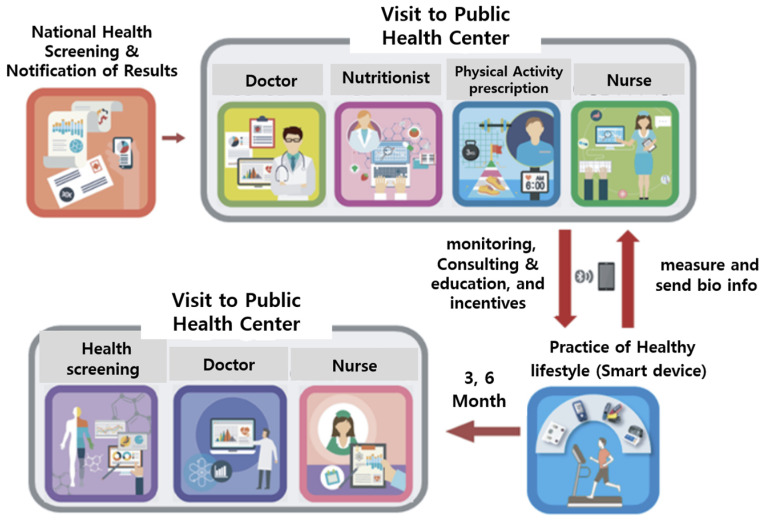
mHealth service process. Source: Korea Health Promotion Institute, 2016 [32].

The service aimed to facilitate health-promoting activities such as walking and self-recording nutrition and exercise using a mobile phone app (Figure 2). The program entailed a six-month service designed to monitor health using mobile apps and devices (activity meter, body composition meter, blood sugar meter, blood pressure meter). Personal health behavior information was collected using pictures and graphs on a daily, weekly, and monthly basis. The expressed figures could be checked on the mobile dashboard. Prior to the commencement of the service, an evaluation was conducted, and tailored health goals were set for each participant to encourage healthy behaviors. The program emphasized nutrition and exercise and offered monthly counseling sessions. The system was designed to carry out missions in accordance with set goals and enabled self-recognition by applying an alarm (push) technique if implementation was not carried out well. Weekly health information was provided through the mobile app, and monthly health reports were provided by comprehensively analyzing individual health status and health behavior information. To sustain participation, various and engaging game techniques such as unexpected missions and commenting were included. Health points were accumulated according to the level of participation, and rewards were given based on the accumulated rank. In the community, participants could support each other through writing and sharing information. After three months, participants revisited the public health center for a follow-up checkup, where goals were reassessed and counseling was adjusted based on changes in measurements. This process was repeated for an additional three months. At the end of six months (24 weeks), a final evaluation was conducted, and the program was concluded.

### 2.3. Analysis Model and Variables

**Figure 3:**
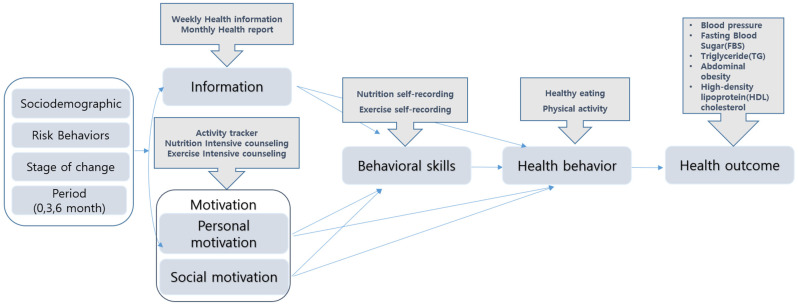
Modified Information–Motivation–Behavioral Skills model of health behavior.

The analytical model of this study was designed by integrating the IMB model of health behavior, as modified in various studies [30,33,34]. The model was constructed to partition the independent and dependent variables of mHealth BCTs based on constructs, in accordance with the theoretical framework (Figure 3).

The dataset encompasses life-log data capable of capturing changes in intervention service behavior, detailed activity logs from a mobile phone app, self-report surveys, and clinical measurements, all linked to personal identification keys and thereby integrated. At this time, the questions in the survey were applied to large-scale national surveys and examinations such as the Korea National Health and Nutrition Examination Survey [35], Community Health Survey [36], and National Dietary Guidelines for Koreans [37].

#### 2.3.1. Dependent Variables

This study examined changes in the number of health risk factors as outcome variables to assess the effect of metabolic syndrome on health improvement. Next, health behaviors were examined, focusing on healthy eating and physical activity. The healthy eating lifestyle practice score ranges from 1 to 9 points, with 1 point assigned for each “Yes” response and 0 points for each “No” response, for the nine items (grain intake, vegetable intake, fruit intake, dairy product intake, balanced meal consumption, breakfast consumption, abstention from animal oil intake, abstention from high-calorie sugar intake such as fried foods, and abstention from salt intake), practiced more than five times in the previous week. The physical activity rate evaluated whether walking or moderate exercise was performed for more than five days in the previous week and was treated as a continuous variable, taking into consideration the duration and intensity of walking and moderate exercise. The data were based on a survey and clinical measurements conducted by mHealth participants over a total of three public health center visits.

#### 2.3.2. Independent Variables Related to mHealth Intervention

Information encompasses knowledge regarding disease etiology, symptoms, treatment procedures, and behavioral practices, as well as technical details enabling the independent acquisition of necessary information [31]. Weekly health information provided through this service included disease-specific insights vital for participants with risk factors for metabolic syndrome, guidance on appropriate physical activities, exercise videos, low-sodium diet cooking tutorials, dietary guidelines, and interpretation of nutritional information. A continuous variable was employed to determine the frequency of checking the weekly health information sent to the participant (1 to 24 times). Subsequently, a monthly report on health status trends (blood pressure, blood glucose, and body fat) and health behavior patterns (achievement rate of goals, exercise duration, cumulative distance traveled, and calorie intake), accompanied by expert opinions, was received for six months. The frequency of utilization (ranging from one to six times) constituted an information variable.

Motivation refers to an individual’s inclination to adopt recommended health behaviors and social support for behavior modification [31]. In this study, the mean number of weekly link days (ranging from zero to seven) was designated as an activity tracker, serving as an indicator of personal motivation by tracking the prescribed exercise regimen. The variables reflecting social motivation included the number of intensive exercise counseling sessions and intensive nutrition sessions conducted by experts (ranging from 0 to 12 sessions).

Behavioral skill is a critical concept that assesses the ability of a highly motivated individual to effectively engage in health behaviors by considering the individual’s objective capacity and self-efficacy in performing a given health-related behavior [31]. In this study, the repetitive completion of food diaries and exercise logs over several days was operationally defined as enhanced behavioral skills, with the average number of recording days per week (ranging from zero to seven) used as a variable.

The control variables included sex, age, education, occupation, city type, presence of national health insurance, smoking status, alcohol consumption status, and behavior change stage, which assessed the readiness of the participant to engage in services. Additionally, longitudinal data comprising the initial, intermediate, and final time variables were employed to observe changes over time.

### 2.4. Statistical Analysis

All intervention items were aligned with the IMB framework. The degree of practice for each construct was then classified through the application of a Group-Based Trajectory Model (GBTM), which constitutes a statistical approach adept at tracing the longitudinal practice patterns of participants, spanning from inception to the conclusion of reiterated, distinct longitudinal datasets. This serves as a valuable tool for characterizing each pattern encounter [38].

The methodology was developed in previous studies [38,39] and is used to model unique trends in the analyzed groups. It categorizes behavior types into groups, over time, and estimates the shape of the trends, for each group. This assumes that over time, mHealth participants will split into different groups that exhibit different types of practices during their time in service. The process of determining the number of practice-type groups has been extensively discussed in prior publications, often involving the selection of an appropriate model using the Bayesian Information Criterion (BIC) [38,39]. A lower BIC value indicates a better-fitting model, and the model with the most negative BIC value is selected. Simultaneously, Akaike’s Information Criteria (AIC) were considered, and the smallest value was prioritized for selection. Nonetheless, it is important to acknowledge that selecting a model with the minimum value may lead to the identification of an undifferentiated group, making it challenging to discern a specific pattern owing to excessive differentiation. In such instances, discretionary judgment assumes significance [40,41,42].

Next, Generalized Estimating Equations (GEE) analysis was conducted. This was employed to estimate causal models across or within panel data. Specifically, it is an analytical method that uses a Generalized Linear Model (GLM) for multivariate variables that do not conform to a normal distribution. The GEE can effectively manage repeated measurement time-series data, which are challenging to handle using a GLM [43,44]. All analyses were conducted using Stata version 16.

## 3. Results

### 3.1. General Characteristics

**Figure 4:**
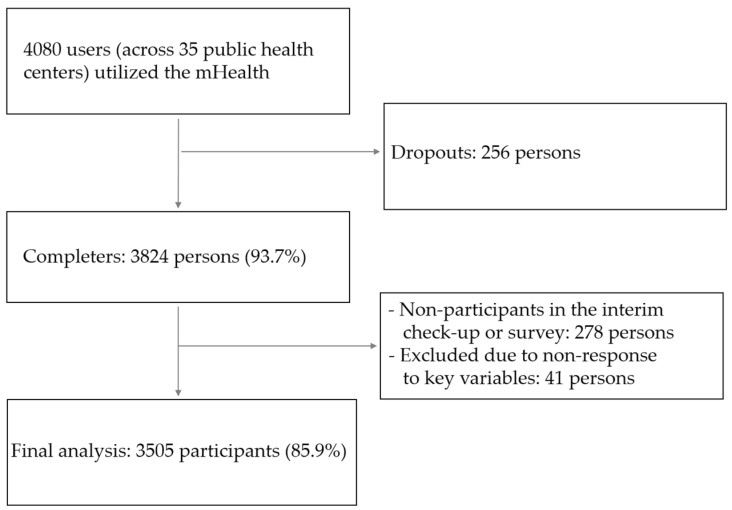
Flowchart of the study participants for this research.

In this study, mHealth participants who completed all surveys and clinical measurements during three visits to public health centers were included. Of the 4080 initial participants, 3505 (85.9%) were included in the analysis, after accounting for dropouts and missing data (Figure 4).

Among them, 44.6% were male and 55.4% were female (Table 1). The highest proportion of participants belonged to the 40s age group (36.0%), followed by the 50s age group (29.4%) and the 30s age group (24.3%). A significant majority (72.0%) of the participants had a college degree or higher education level. The largest occupational category was office workers (30.5%), followed by homemakers/unemployed (23.0%) and managers/professionals (22.5%). Most health insurance subscribers were employed. Regarding municipalities, 40.8% resided in a large city, 42.6% in a small- to medium-sized city, and 16.6% in a rural area. The prevalence of smoking was 12.6%, and the monthly alcohol consumption rate was 59.7%. Prior to participation in the service, it was crucial to assess the willingness of participants to engage. Of the participants, 25.2% were already practitioners (action and maintenance) in the healthy eating change stage, 59.6% were in the preparation stage to commence within one month, and 15.2% were in the pre-preparation stage. Regarding the exercise change stage, 42.1% were already practicing (action and maintenance), 44.2% were in the preparation stage and aimed to initiate within one month, and 13.7% were in the pre-preparation stage. In addition, 57.7% of subjects had one or two health risks, and 42.3% had three or more metabolic symptoms.

### 3.2. Practice Patterns of mHealth Intervention

**Figure 5:**
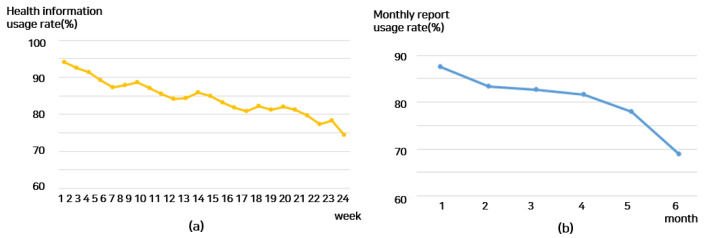

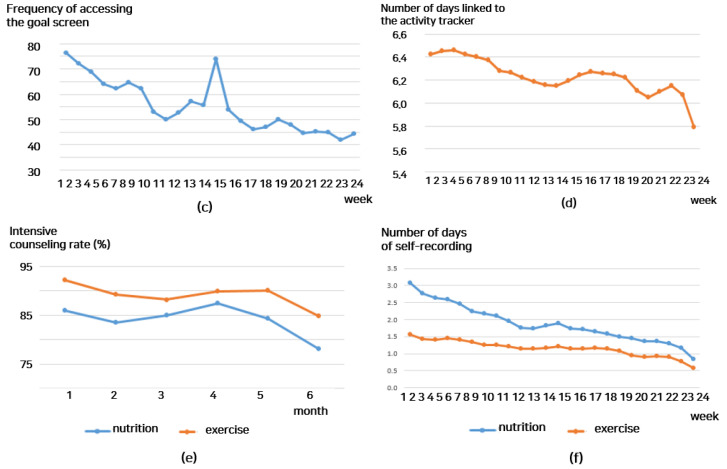
Usage patterns of mHealth interventions over 24 weeks by subject: (**a**) weekly health information utilization rate; (**b**) monthly report utilization rate; (**c**) weekly goal screen access frequency; (**d**) weekly activity tracker access count; (**e**) monthly intensive counseling rate; (**f**) weekly self-recording days.

The usage trends of each intervention among mHealth recipients over the six-month (24-week) period were as follows (Figure 5): The health information usage rate was 94.1% in week 1 but decreased to 86.0% in the 12th week and 74.4% in the 24th week. The monthly report rate was maintained from 87.6% at one month to 82.6% at three months but decreased to 68.9% at six months. Next, the weekly frequency of accessing the goal screen, which tracks various activity metrics (such as steps, calories burned, exercise time, and moderate exercise), was examined. It decreased slightly from 76.3 times at week 1 to 55.8 times at week 12 and then to 74 times at week 13 and to 44.4 times at week 24. The number of days linked to the activity tracker remained at 6.4 days at week 1 and 6.1 days at week 12 but decreased to 6.1 days at week 23 and 5.8 days at week 24. Participation in intensive nutrition counseling, held once a month, decreased to 86% at one month, 84.9% at three months, and 78.1% at six months. Participation in intensive exercise counseling was 92.1% at one month, 88.2% at three months, and 84.8% at six months. Meanwhile, examining nutrition self-recording and exercise self-recording, nutrition self-recording was 3.1 days at week 1, 2.8 days at week 2, 1.8 days at week 12, 1.2 days at week 23, and tended to decrease to 0.8 days at week 24. Exercise self-recording gradually decreased to 1.6 days at week 1, 1.2 days at week 12, and 1.0 days at week 19 and then decreased to 0.8 days at week 23 and 0.6 days at week 24.

### 3.3. Validation of the Analytical Model and Practice Patterns 

The GBTM categorizes longitudinal behavioral patterns over time, using repeated measurements. Following the examination of the BIC and AIC values, two group classifications were deemed most appropriate. However, to investigate additional differentiation patterns through the application of the GBTM, the number of groups was expanded to a level where the disparity in the BIC size was minimal. Simultaneously, a secondary assessment was conducted to ascertain the presence of statistical differences between the groups using mean difference tests (T-test, analysis of variance) and regression analysis.

Finally, a fitting model for each IMB construct was selected and classified into three categories: information, motivation, and behavioral skills (Table 2).

### 3.4. Practice Patterns by IMB Components

#### 3.4.1. Information

**Figure 6:**
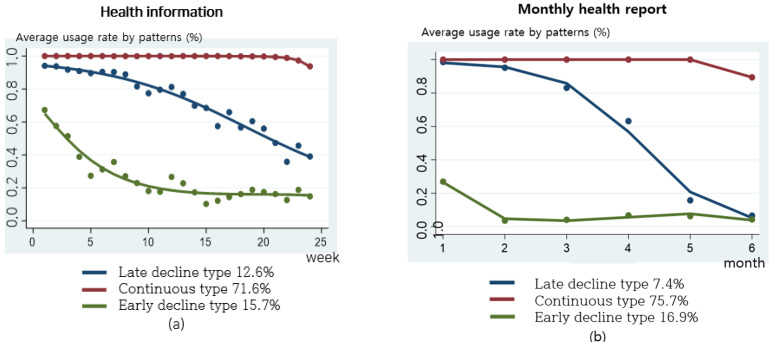
Practice patterns for health information and monthly reports: (**a**) weekly health information usage rate by practice patterns; (**b**) monthly report usage rate by practice patterns.

Health information and monthly reports were categorized into three types (Figure 6). The types of health information checking are as follows: the continuous type, 71.6%; the late decline type, 12.6%; and the early decline type, 15.7%. The monthly report showed the continuous type, 75.7%; the late decline type, 7.4%; and the early decline type 16.9%.

#### 3.4.2. Motivation

(1) Personal motivation

**Figure 7:**
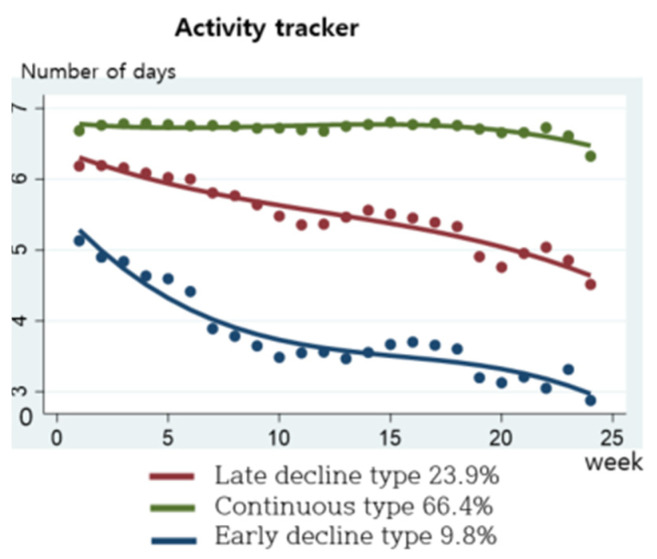
Practice patterns for activity tracker.

The mHealth service recommends linking with the activity tracker mobile phone app at least five times a week (Figure 7). From the results of categorizing weekly interlocking practices, 66.4% were the continuous type, linking an average of seven times a week; 23.9% were the late decline type, practicing an average of five times per week and then gradually decline; and 9.8% were the early decline type.

(2) Social motivation

**Figure 8:**
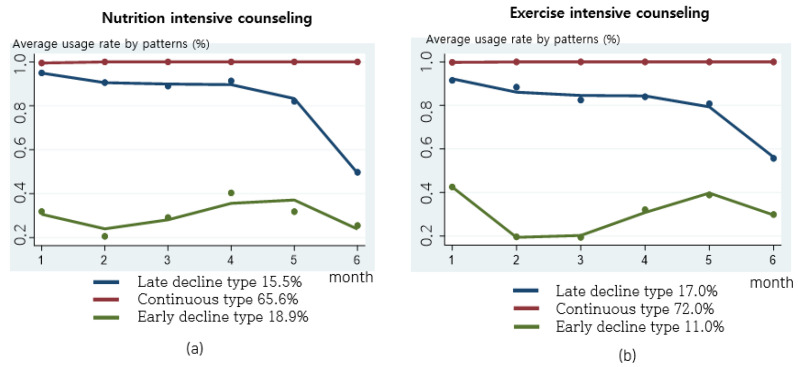
Practice patterns for nutrition and exercise intensive counseling: (**a**) monthly nutrition intensive counseling rate by practice patterns; (**b**) monthly exercise intensive counseling rate by practice patterns.

Nutrition intensive counseling and exercise intensive counseling were provided once a month (Figure 8). The distribution of nutrition intensive counseling practice was as follows: 65.6% for the continuous type, 15.5% for the late decline type, and 18.9% for the early decline type. For exercise intensive counseling, the distribution was 72.0% for the continuous type, 17.0% for the late decline type, and 11.0% for the early decline type. For the practice patterns of intensive counseling, the late decline type started to decline at five months. For the early decline type, practice remained low for two to three months. For the early decline type, practice remained low for two to three months, and after intermediate face-to-face counseling (three months), intensive counseling increased at four to five months and then decreased again.

#### 3.4.3. Behavior Skills

**Figure 9:**
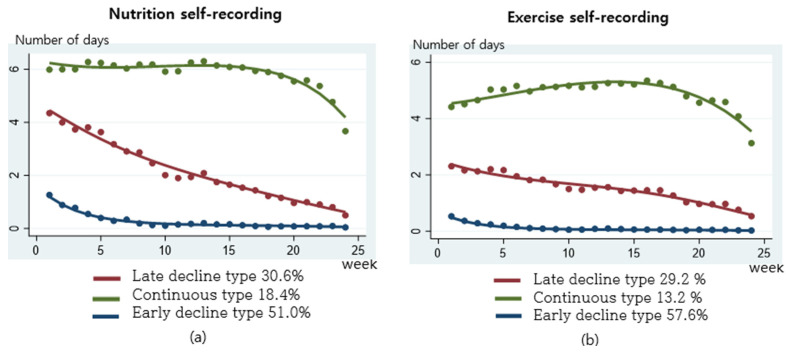
Practice patterns for nutrition and exercise self-recording: (**a**) weekly nutrition self-recoding rate by practice patterns; (**b**) weekly exercise self-recoding rate by practice patterns.

Behavior skills were assessed using weekly nutrition self-recording and exercise self-recording (Figure 9). Nutrition self-recording was 18.4% for the continuous type, 30.6% for the late decline type, and 51.0% for the early decline type. Exercise self-recording was 13.2% for the continuous type, 29.2% for the late decline type, and 57.5% for the early decline type. Compared to other practices, the early decline type was the highest, followed by the late decline type and the continuous type.

### 3.5. Health Risk Factors and Health Behavior 

To examine changes in health risks and health behaviors, mHealth participants were analyzed based on clinical information and self-reported questionnaires measured during initial, mid-term, and final visits to public health centers (Table 3). Examining changes in health risk factors, the initial mean decreased from 2.4 to a mid-term mean of 1.7 and subsequently to a final mean of 1.4. The proportion of subjects with three or more metabolic syndrome risk factors declined from 42.3% in the initial period to 27.2% in the mid-term and further to 19.2% in the final period. Additionally, by the end of this study, 24.5% of the subjects exhibited no health risk factors. Furthermore, the analysis of health behaviors revealed improvements in healthy eating and physical activity. The healthy eating lifestyle practice score increased from an initial 5.0 points to 6.4 points by the final measurement. The physical activity rate also showed significant improvement, rising from 41.5% at the beginning of this study to 59% at its conclusion.

### 3.6. Health Promotion Effects by Practice Patterns of mHealth 

A multi-GBTM methodology was applied. Interventions were integrated by construct (information, motivation, and behavioral skills) to distinguish the quantified practice patterns of participants. In addition, the effect on improving healthy living practices and health outcomes according to the three practice types of information–motivation–behavioral skills (the continuous type, the late decline type, and the early decline type) was examined. The analysis was conducted using the GEE of panel regression analysis that considers the correlation of response variables resulting from three repeated measurements: before, during, and after. The control variables included sex, age, education, occupation, city type, risky behaviors (smoking and alcohol consumption), stage of behavior change (continuous variable), and service participation period (Table 4).

In Model 1, the results showed that the continuous type of information (β = 0.217, *p* < 0.001), the continuous type of personal motivation (β = 0.156, *p* < 0.001), and the continuous type of behavioral skills (β = 0.156, *p* < 0.001) all significantly influenced the adoption of healthy eating. In Model 2, when analyzing the effects of information, motivation, and behavioral skills on physical activity, it was found that there was a significant impact from the continuous type of behavioral skills (β = 0.165, *p* < 0.001). Model 3 examined the effect of health-related behavior practice (healthy eating and physical activity) on health risk factors. The results showed that the higher the healthy eating score (β = −0.021, *p* < 0.001), the more physical activity significantly reduced health risk factors (β = −0.072, *p* < 0.001). Model 4 examined the impact of changes in the number of health risk factors by considering all variables of information, motivation, behavioral skills, and health behavior. The results showed that health risk factors decreased as the continuous type of behavioral skills (β = −0.116, *p* < 0.001) and health behavior practice (healthy eating, β = −0.019; physical activity, β = −0.067) increased.

The other control variables showed that compared to males, females engaged in less physical activity but were better at practicing healthy eating. As age increased, healthy eating improved. The physical activity rate was high in those in their 20s and 60s, with the lowest physical activity rate found in those in their 40s. Smoking and alcohol consumption had a negative effect on healthy eating. Alcohol consumption reduced physical activity, and smoking had a negative effect on health risk factors. The higher the stage of behavior change and the longer the period of participation (three months → six months), the more positive the influence on health outcomes; however, the number of health risks increased with age.

## 4. Discussion

### 4.1. Principal Findings

The IMB model can be effectively utilized to design and evaluate various interventions by incorporating strategies related to information, motivation, and behavior skills within each construct. Interventions based on IMB theory aimed at improving medication compliance, self-management, and risk prevention behavior have demonstrated strong efficacy [31,33,34,45,46,47]. This study applied the IMB theory to mHealth intervention services to improve the risk factors for metabolic syndrome and examined its effects and influencing factors. Among the IMB components, the higher the continuity of behavioral skills and rate of health behavior practice, the more positive the influence on improving health. This finding is consistent with those of systematic reviews and meta-analyses that have shown that physical activity and lifestyle interventions based on mHealth technology are effective in reducing cardiometabolic risk [8].

Although not presented in Table 4, an examination of the relationship between IMB components reveals that the late decline type and continuous types of information and motivation influence behavioral skills and health behavior, aligning with existing research [31]. In Model 1, healthy eating habits were implemented in all continuous types of IMB other than social motivation. A separate detailed analysis showed that social motivation had an effect on healthy eating habits, but its influence was smaller than that of information or personal motivation. Nevertheless, the participants were already receiving social support, such as receiving information from experts, and the difference here can be seen that participants with high personal motivation who continuously put into practice the information provided were practicing healthy eating well. In Model 2, it was confirmed that only continuous types of behavioral skills affect physical activity. It can be assumed that IMB practice needs to be strengthened more to change physical activity than to change healthy eating. Next, in a separate detailed analysis, it was confirmed that IMB affects health behavior, but in Models 3 and 4, only health behavior (healthy eating, physical activity) was confirmed to be significant for health outcomes (reduction in health risk factors). This appears to have canceled out the individual effects of IMB when considering all variables. However, it is important to consider all components because IMB must be performed well for health behavior to continue.

In this study, participants learned about the activities and methods necessary for health management through mHealth services, and their self-monitoring and goal management abilities improved with the use of an activity tracker. Counseling that assists in changing behavior through social motivation may positively affect behavioral skills. Moreover, the continuous application of behavioral skills has a decisive influence on the performance of health behaviors. Behavioral skills are a critical concept that assesses whether a motivated individual can proficiently execute health behaviors using available health information. They center on an individual’s objective capability and self-efficacy in performing specific health-related behaviors. The behavioral skills in this study involved repeated self-recording of nutrition and exercise, which are difficult to practice. As seen in the practice pattern of weekly nutrition and exercise self-recording in Figure 9, the percentage of the continuous type in behavioral skills was lower than that of other behavioral change techniques (BCTs), with more than 50% showing an early decline type. In other words, practicing behavioral skills was more difficult than other interventions, but those who practiced them well had a strong willingness to continue participating in mHealth services, which appeared to have a positive effect on health outcomes.

According to a qualitative study on change in health behavior and app use experience [48], the act of recording behavior via apps engenders a sense of control, indicating proficient self-management of health. This self-awareness in effectively managing health facilitates the implementation of health behavior changes. This finding aligns with the results indicating that participants initiate self-management by actively acknowledging their attitudes and behaviors towards health through self-monitoring and behavioral feedback techniques. Furthermore, self-management skills were enhanced through persistent repetition, as evidenced in previous studies [48,49].

In this study, in addition to the IMB composition, it was difficult to quantitatively examine changes in daily life expected to result from positive behavioral changes, such as purchasing healthy food, joining an exercise group, learning a new sport, and purchasing exercise equipment. However, in focus group interviews that analyzed the experiences of mHealth participants in a prior study [50], participants who had health improvement effects continued to make choices that were beneficial to their health. In other words, practicing health behaviors and experiencing health improvements would have increased self-efficacy to continue health-seeking behaviors in daily life.

However, mHealth faces challenges in the management of metabolic syndrome. Although mHealth has shown health improvement effects in individuals with risk factors for metabolic syndrome, it also has limitations owing to the lack of compulsion in non-face-to-face interactions. Mobile phone apps operate non-face-to-face, where users transmit practice information, and counseling and management are conducted based on the degree of participation. Therefore, if inactive participants do not input their practice information, counselors find it difficult to understand their behavior and can only provide general and simplistic information. Consequently, inactive participants lose interest in mobile phone apps and do not engage in health practices, creating a vicious cycle. Another study [51] points out that non-users of mobile phone apps avoid them because the mobile phone platform does not fit their lifestyle patterns or because the activity of manually entering health practices is cumbersome. This suggests that although mobile phone platforms have several advantages in a non-face-to-face environment, face-to-face or similar interventions are still needed.

Future studies should compare and analyze the sociodemographic characteristics between continuous-type and non-continuous-type subjects. Additionally, interventions incorporating behavioral economic factors using nudges should be developed and tailored to subjects through the design of an automated algorithm.

### 4.2. Limitations and Implications

This study had several limitations, but it has implications nonetheless. First, the lack of a comparison group made it impossible to control for external variables, thereby limiting causal inferences. In the evaluation of program interventions, randomized controlled trials or studies using quasi-experimental methodologies are typically conducted to compare an experimental group with a control group and explain the causal effects. A previous study used quasi-experimental methods, such as propensity score matching analysis and difference-in-differences analysis, to compare the effectiveness of the mHealth intervention with a control group. The results indicated that the mHealth intervention group was more effective than the control group [52]. Second, although the aim was to examine various practice types using GBTM, participants maintained a similarly high adherence rate because of personalized management. Therefore, the practice types were not diverse, posing limitations in explaining the full spectrum of practice changes over time. Nonetheless, unlike most intervention studies that derive causal results through before–after cross-sectional analysis at two or three time points, this study is meaningful in quantifying individual practice patterns over time according to the concepts of health behavior theory. This can be utilized as a basis for determining the selection of intervention elements and frequency of interventions in future mHealth services. Third, when selecting subjects, there is a possibility that the majority of users will participate voluntarily rather than in an experimental setting for research, resulting in selective bias. This can reduce internal validity in revealing the causal relationship between services. Nevertheless, looking at the characteristics of the subjects analyzed in this study, compared to the voluntary use of public health center users, 58.7% of users who received follow-up health care services due to abnormal findings after the national health checkup were randomly included, which can be explained by data minimizing user selection bias.

Despite the limitations noted above, this study employed large-scale mHealth empirical data to categorize practice types according to health behavior theory and evaluate their health promotion effects. Unlike typical intervention studies that rely on self-report surveys and clinical measurements for assessing behavioral changes, this study utilized life-log data to capture nuanced behavioral patterns over time. This methodological approach enhances the depth and reliability of our findings.

## 5. Conclusions

Based on our comprehensive analysis using a multi-GBTM methodology, integrated interventions focusing on information, motivation, and behavioral skills delineated distinct practice patterns among participants. Our study identified significant influences from continuous types of these constructs, notably enhancing the adoption of healthy eating and physical activity. These findings underscore the critical role of targeted interventions in promoting healthier lifestyles and reducing health risk factors associated with metabolic syndrome.

## Figures and Tables

**Table 1:** Characteristics of the study participants (No. of individuals: 3505).

Analyzed Variables		N	%	Analyzed Variables		N	%
Sex	Male	1562	44.6	Smoking	Yes	442	12.6
					No	3063	87.4
	Female	1943	55.4	Alcohol consumption (monthly)	Yes	2092	59.7
					No	1413	40.3
Age group	20s	161	4.6	Stages of change in healthy eating	Precontemplation	97	2.8
	30s	853	24.3		Contemplation	435	12.4
	40s	1263	36		Preparation	2088	59.6
	50s	1031	29.4		Action	352	10
	60s	197	5.6		Maintenance	533	15.2
Education level	High school graduate or less	981	28	Stages of change in exercise	Precontemplation	155	4.4
	College degree or higher	2524	72		Contemplation	325	9.3
Occupation	Managers/professionals	788	22.5		Preparation	1549	44.2
	Office workers	1070	30.5		Action	707	20.2
	Service/sales workers	492	14		Maintenance	769	21.9
	Other workers	349	10	No. of health risk factors	Mean (standard deviation)	2.4 (1.1)	
	Homemakers/unemployed	806	23				
Municipality	Large city	1430	40.8		One	950	27.1
					Two	1072	30.6
	Small- to medium-sized city	1492	42.6		Three	835	23.8
					Four	508	14.5
	Rural area	583	16.6		Five	140	4

**Table 2:** BIC and AIC values for each component concept.

Analyzed Variables		Group	BIC † (N = 3505)	AIC (N = 3505)	Group1 (%)	Group2 (%)	Group3 (%)
Information	Multi-information	Two	−24,516.42	−24,464.04	74.1	25.9	
		Three	−2206.72	−21,986.62	69.7	16.3	14.0
Motivation	Activity tracker	Two	−125,497.55	−125,466.74	75.7	24.3	
		Three	−122,140.39	−122,094.18	66.4	23.9	9.8
	Integrated intensive counseling	Two	−11,009.87	−10,957.50	69.3	30.7	
		Three	−9910.26	−9830.16	16.9	71.4	11.7
Behavior Skills	Integrated self-recording	Two	−198,589.07	−198,530.53	34.3	65.6	
		Three	−188,488.45	−188,402.19	18.3	33.3	48.4

† Based on the minimum BIC value, two groups are deemed appropriate. However, to explore differentiation comprehensively, significant differences between groups were determined based on practice type for each constituent concept, ultimately resulting in the division into three groups.

**Table 3:** Changes in health risk factors and health behavior according to participation period (No. of individuals: 3505).

Variable		Initial		Intermediate (3 Months)		Final (6 Months)		Intermediate Change (%p)	Final Change (%p)	F
		N	%	N	%	N	%			
No. of health risk factors	Mean (standard deviation)	2.4 (1.1)		1.7 (1.3)		1.4 (1.2)		−0.7	−1	557.77 *
	None	-	-	644	18.4	859	24.5	18.4	24.5	
	One	950	27.1	1009	28.8	1134	32.4	1.7	5.3	
	Two	1072	30.6	898	25.6	840	24	−5	−6.6	
	Three	835	23.8	593	16.9	476	13.6	−6.9	−10.2	
	Four	508	14.5	298	8.5	174	5	−6	−9.5	
	Five	140	4	63	1.8	22	0.6	−2.2	−3.4	
Healthy eating score † Mean (standard deviation)		5.0 (2.1)		6.0 (1.9)		6.4 (1.9)		1	1.4	448.22 *
Physical activity rate ††	walking and moderate exercise	324	9.2	421	12	493	14.1	2.8	4.9	91.91 *
	moderate exercise	76	2.2	49	1.4	42	1.2	−0.8	−1	
	walking	1056	30.1	1439	41.1	1532	43.7	11	13.6	
	do nothing	2049	58.5	1596	45.5	1438	41	−13	−17.5	

* *p* < 0.001. † Healthy eating score is divided into 9 items consumed at least 5 times a week (frequency of grain intake, frequency of vegetable intake, frequency of fruit intake, frequency of dairy product intake, frequency of balanced meals, frequency of breakfast, non-consumption frequency of animal oil, non-consumption frequency of high-calorie foods such as fried foods, non-consumption frequency of salt) added together. †† Physical activity rate means walking for more than 10 min five times a week or engaging in moderate-intensity exercise for more than 10 min.

**Table 4:** Impact on health behavior and health risk factors by practice patterns of mHealth (No. of observations: 10,515; No. of individuals: 3505).

Analyzed Variables			Model 1		Model 2		Model 3		Model 4	
			Practice Patterns of I,M,B → Healthy Eating		Practice Patterns of I,M,B → Physical Activity		Health Behavior → Health Risk Factors		All → Health Risk Factors	
			β	S.E	β	S.E	β	S.E	β	S.E
Sociodemographic characteristics		Sex (ref = Male)								
		Female	0.250 ***	−0.053	−0.040 *	−0.017	−0.475 ***	−0.04	−0.482 ***	−0.04
		Age Group (ref = 20s)								
		30s	0.286 **	−0.11	−0.122 ***	−0.035	0.242 **	−0.084	0.259 **	−0.084
		40s	0.775 ***	−0.109	−0.201 ***	−0.035	0.358 ***	−0.082	0.394 ***	−0.083
		50s	1.147 ***	−0.111	−0.142 ***	−0.036	0.452***	−0.084	0.492 ***	−0.085
		60s	1.634 ***	−0.141	−0.032	−0.045	0.454 ***	−0.106	0.503 ***	−0.107
		Education (ref = High school graduate or less)								
		College degree or higher	0.057	−0.054	−0.049 **	−0.017	−0.118 **	−0.041	−0.113 **	−0.041
		Occupation (ref = managers/professionals)								
		Office workers	−0.139 *	−0.06	−0.055 **	−0.019	−0.068	−0.046	−0.063	−0.046
		Service/sales workers	−0.211 **	−0.075	−0.004	−0.024	0.091	−0.057	0.086	−0.057
		Other workers	−0.190 *	−0.085	0.032	−0.027	−0.084	−0.065	−0.065	−0.065
		Housewives/unemployed	−0.155 *	−0.07	−0.024	−0.022	0.073	−0.053	0.09	−0.053
		Municipality (ref = Bic city)								
		Small- to medium-sized city	−0.02	−0.048	−0.056 ***	−0.015	−0.04	−0.036	−0.028	−0.036
		Rural area	−0.104	−0.063	−0.167 ***	−0.02	0.003	−0.048	0.008	−0.048
Risk behaviors		Smoking (ref = No) Yes	−0.336 ***	−0.066	0.023	−0.022	0.159 ***	−0.047	0.147 **	−0.047
		Monthly alcohol consumption (ref = No) Yes	−0.208 ***	−0.041	−0.067 ***	−0.014	−0.016	−0.028	−0.017	−0.028
Stages of behavior change		Stages of change in Healthy eating (Precontemplation = 1, continuous variable)	0.612 ***	−0.017	0.035 ***	−0.006	−0.034 **	−0.011	−0.045 ***	−0.011
		Stages of change in exercise (Precontemplation = 1, continuous variable)	0.080 ***	−0.017	0.133 ***	−0.006	−0.049 ***	−0.011	−0.034 **	−0.011
Participation period		(ref = Initial)								
		Intermediate (3 months)	0.727 ***	−0.032	0.091 ***	−0.011	−0.585 ***	−0.02	−0.588 ***	−0.02
		Final (6 months)	0.976 ***	−0.032	0.120 ***	−0.012	−0.856 ***	−0.021	−0.860 ***	−0.021
Integrated information		(ref = Early decline type)								
		Late decline type	0.089	−0.083	0.037	−0.027			0.064	−0.063
		Continuous type	0.217 *	−0.085	0.021	−0.027			−0.034	−0.065
Motivation	Personal	Activity tracker (ref = Early decline type)								
		Late decline type	0.055	−0.083	0.004	−0.026			−0.029	−0.063
		Continuous type	0.156 *	−0.08	0.043	−0.025			−0.097	−0.06
	Social	Integrated Intensive counseling (ref = Early decline type)								
		Late decline type	−0.063	−0.088	−0.049	−0.028			0.016	−0.067
		Continuous type	−0.105	−0.094	−0.013	−0.03			0.021	−0.071
Behavior skills		Integrated Self-recording (ref = Early decline type)								
		Late decline type	0.015	−0.051	0.011	−0.016			−0.023	−0.039
		Continuous type	0.156 *	−0.066	0.165 ***	−0.021			−0.116 *	−0.05
Health behavior		Healthy eating					−0.021 **	−0.006	−0.019 **	−0.006
		Physical activity					−0.072 ***	−0.018	−0.067 ***	−0.018
Intercept			1.919 ***	−0.165	0.115 *	−0.053	2.767 ***	−0.11	2.800 ***	−0.122

* *p* < 0.05, ** *p* < 0.01, *** *p* < 0.001

## Data Availability

We kindly request that any inquiries regarding the data used in this study be directed to the corresponding author. The datasets used in this study are available upon request.
